# The World’s Columbian Exposition

**Published:** 1892-01

**Authors:** 


### The World's Columbian Exposition.

Quite a great deal is being said and printed about the World's Fair, but when we think of the magnitude of this undertaking and of how many thousands are interested in it, not only in this country but in nearly every other country on the face of the globe, we are astonished that we do not hear and read more about it.

First of all we will notice that it is at the present moment giving employment to many who might otherwise be suffering for want of work, and when we hear that from thirty-five to forty car-loads of construction material are arriving daily, this alone gives us some idea of the rapidity with which the buildings are rising, but if we try to imagine what those buildings will contain when completed, it baffles us altogether; and all we can say is, we know no one will go away disappointed, for there will be such a multiplicity of things both interesting and instructive for every man, woman and child who may have the privilege of visting the World's Columbian Exposition.

C. W. Wynkoop, who has been sent out by a London syndicate to find the gold mines in the Biblical lands of Ophir, where King Solomon and the Queen of Sheba got their riches, will make a report of his investigations, and promises to furnish some interesting matter for the mining department.

Major John Wilson, of Auckland, New Zealand, has submitted a proposition to the Foreign Affairs Committee to bring a colony of Maoris to the Exposition, house them in their native-built forts, and let them show their native costumes, home-life and methods of warfare. The proposition is regarded with some favor as it would add greatly to the valne of the general ethnological exhibit of the Exposition.

Then we understand that Columbia, S. A., will celebrate the Anniversary of its Independence by opening an Exposition on July 20th, 1892, which will continue until the end of October. It will embrace an extensive showing of the resources and products of the country, also a historical, archæological and ethnoloigical exhibit. At the close of this Exposition the best portion of the collection will be sent to the World's Columbian Exposition as part of the exhibit from the Republic of Columbia.

British Columbia has decided to build a structure which will be a novelty in architecture. It will be composed of every variety of wood known to the forests of that region. The building will consist of sections of contrasting woods neatly mortised together. The roof will be of native slate and a variety of cedar shingles. It is intended to ship the building in sections ready to be erected on its arrival.

And now while we are mentioning a few things that others are doing to add to the interest and attractiveness of this great enterprise, we may well ask the question, What is our own department doing? From letters received we know that great zeal and enthusiasm are manifested among the leading dentists of every State, and they are working hard to make their department as attractive to their professional brethren in this country. and those who shall visit them from distant lands, as well as to all interested in every thing concerning the preservation of the teeth, etc., etc., as any other department. Much useful information is being gathered concerning the profession and practice of dentistry in every State of the Union, and we hope to have a very large exhibit of instruments and appliances that were used in former times, both to torture and relieve, and also many of the best and latest inventions in use at the present time, and by a comparison of the two we shall be the better able to judge of what science has done for us in the last two centuries at least, and no doubt shall find the improvements in dental apparatus and workmanship have kept pace with those of all other trades and professions. It is with pleasure we note the unity of purpose evidenced in the letters received from all the States, east as well as west, and with true pride in this department, predict for it a grand success. E. B.

